# Effects of Aging on the Obstacle Negotiation Strategy while Stepping over Multiple Obstacles

**DOI:** 10.1038/s41598-018-26807-5

**Published:** 2018-06-05

**Authors:** Jung Hung Chien, Jerod Post, Ka-Chun Siu

**Affiliations:** Physical Therapy Education, University of Nebraska Medical Center, 984420 Nebraska Medical Center, Omaha, 68198 Nebraska USA

## Abstract

Forty-seven percent of falling accidents in older adults are caused by tripping over obstacles. Understanding what strategies are involved in obstacle negotiation in older adults could reduce fall risks. There is a paucity of research investigating how healthy adults negotiate multiple obstacles, which may better reflect the complexity of the real environment. The presence of a second obstacle has induced mixed results in the obstacle negotiation of healthy older adults with the interval between obstacles two steps apart. This study extended the knowledge to understand what strategies healthy younger and older adults used to step over two obstacles placed at three-step-length apart. Twenty healthy subjects performed 2 tasks: level ground walking and stepping over two obstacles. The height of each obstacle was set at 10% of subjects’ leg height. We found that aging significantly increased the toe clearance in leading and trailing legs when stepping over the obstacles at a three-step-length interval. Toe clearance was higher while stepping over the second obstacle than the first one in older adults. These results had two-fold meanings: the three-step-length interval was long enough to trigger the adjustment of the obstacle negotiation strategy, and aging led older adults to use conservative negotiation strategies.

## Introduction

Falls are serious health problems for older adults. One-third of Americans over the age of 65 experience a fall at least once per year^1–3^. Specifically, forty-seven percent of falling accidents are caused by tripping over obstacles^1,4^. These falls lead to more than 2.8 million injuries annually, and a total cost reaching $3.4 billion, including 800,000 hospitalizations and more than 27,000 deaths^5^. Thus, understanding what strategies are involved in obstacle negotiation in older adults has become an important undertaking.

The age-related effect on “single” obstacle negotiation has been well documented^6–13^. The most consistent observation across studies comparing healthy older to younger adults is a significant increase in the elevation of the swing leg over an obstacle in an effort to maintain a safe elevation distance between the swing foot and the obstacle^6–9^. Further, a shorter step length, an altered toe-off-to-obstacle interval (horizontal distance from the obstacle to the location where toe off) before stepping over the obstacle, and a shorter obstacle-to-heel strike interval (horizontal distance from the obstacle to the location where heel strike) after stepping over the obstacle have also been observed in older adults^7–9,11^. From a biomechanical point of view, a shorter step length induces a slower walking speed in older adults while negotiating the obstacle, and a slower walking speed ensures minimal requirement for torso momentum to maintain stability of the upper body^8^. At the same time, large hip rotation angles help an older adult elevate the leg high enough to step over the obstacle^6,7,14^.

These changes of gait kinematics in older adults might be due to the deterioration of sensory (visual, proprioceptive, and vestibular) systems^12,13,15^, and the reduction of muscle strength^16^. The deteriorations in vision, proprioceptive, and visual-spatial cognition leads to the adoption of conservative strategies in older adults (shorter step length, slower walking speed, shorter obstacle-to-heel strike interval, smaller knee joint flexion and greater ankle dorsiflexion when heel-strike of leading leg after stepping over the obstacles, larger hip joint flexion at the moment of passing the obstacle) to allow for increased control of the upper torso^12,13,15^. Moreover, muscle weakness has a negative impact on mobility, significantly decreasing push-off power^16^.

From a neuro-physiological point of view, these biomechanical changes while negotiating an obstacle in older adults could be due to the attenuation of executive function^17^. Efficient obstacle elevation requires high cognitive processes such as strategically planning to step over obstacles through vision^18^, the real-time monitoring of changes in the environment, and the adjustment of future actions using proprioceptive feedback from the trailing leg^19^. These functions can be reduced in older adults due to the deterioration of the prefrontal cortex^20^, which is involved in decision making and the controlling and planning of complex cognitive behavior. Therefore, older adults need more time to negotiate the obstacles than young adults. In addition, this deterioration of executive function has been related to the reductions in step length^21^.

Regardless of the alteration of strategies involved in obstacle negotiation in older adults reported in previous studies, most healthy older adults still can successfully complete obstacle negotiation without tripping over the obstacle. Successful obstacle negotiation requires that one leg steps over the obstacle first (leading leg), followed by the other leg (trailing leg). The trajectory of the leading leg differs from the trajectory of trailing leg. In addition, the maximum elevation of the trailing leg, which cannot be aided by vision, is lower than that of the leading leg^10^. Two hypotheses have been suggested to explain this phenomenon: (1) vision pre-programming^18^ and, (2) unilateral and bilateral transfer^19^. Although visual information is absent as the trailing leg steps over the obstacle, the trailing leg can successfully negotiate the obstacle without tripping. It is likely that vision plays an important role in recognizing the shape and height of obstacles for the take-off location and trajectory of the trailing leg to be determined in advance^18^. Meanwhile, the somatosensory sensory system plays a crucial role in transferring the height and shape information from the leading to the trailing leg^19^.

Surprisingly, all of the aforementioned studies only focus on “single” obstacle negotiation. However, investigating the effect of age on single obstacle negotiation may not reflect the complexity of the real world. To the best of our knowledge, there are only four existing studies that attempt to understand how humans negotiate multiple obstacles^8,22–24^. Krell and Patla indicated that the toe-off-to-obstacle distance of the trailing leg is related to the distance between the two obstacles in young adults^22^. If only one-step length between two obstacles is allowed, the toe-off-to-obstacle distance is shortened significantly while stepping over the second obstacle^22^. On the other hand, if two-step length is allowed, the toe-off-to-obstacle distance over the second obstacle shows no difference in comparison to that over the first^22^. However, if the relative distance between two obstacles is longer than two-step length, the toe-off-to-obstacle distance is shortened again. This previous study confirms that vision information can be used in the pre-programming of the trajectory of the trailing leg by around two steps away. In a follow-up study, a significantly decreased toe clearance in the trailing leg is observed in healthy young adults while stepping over the second obstacle in comparison to the first^23^. This result infers that young adults could learn the shape and height of obstacles from stepping over the first obstacle and then use this learned experience for stepping over the second obstacle.

While investigating the effect of aging on multiple obstacle crossing, there is only a significant aging effect on placing the heel of the leading leg closer to the obstacle in older adults than in young adults after stepping over the obstacle^8^. However, the presence of the second obstacle does not affect the obstacle negotiation strategy in young and older adults when two-step length between obstacles is allowed^8^. A recent study that investigates the negotiation of multiple obstacles in healthy older adults and in patients with Parkinson’s disease, indicates that the presence of a second obstacle increases stride duration in both groups when two-step length between obstacles is provided^24^. The rationale is that both healthy older adults and patients with Parkinson’s disease require more time to plan and execute adjustments due to the presence of the second obstacle, to ensure success of obstacle negotiation^24^. These four studies have already presented mixed results that clearly highlight the importance of continued research in the area of multiple obstacle negotiation, specifically when the intervals between multiple obstacles are different.

In the current study, we extended the understanding of the effect of aging on multiple obstacle negotiation strategies when an interval of three-step-length between two identical obstacles was allowed. By using this method, the leading and trailing legs would be altered in order to step over two obstacles. We also attempted to answer these questions: (1) Would age affect kinematics (i.e. joint angles and spatial gait parameters) while stepping over obstacles? (2) Would obstacle negotiation strategies be similar between the first obstacle and the second obstacle? (3) Would an interaction effect between age and obstacle exist while negotiating two obstacles? Four obstacle negotiation events were investigated to address above questions: the moment when both the leading and trailing leg crossed the obstacle, the moment when the heel of leading leg contacted the floor (heel-strike) after stepping over the obstacle, and the moment when the toe of the trailing leg lifted off the floor (toe-off) before stepping over the obstacle. The dependent variables were the spatiotemporal gait kinematics at these four events. Each spatial gait parameter was normalized to leg length to account for between-subjects differences. We hypothesized that: (1) older adults would increase the toe clearance in both legs and decrease the obstacle-to-heel strike distance of the leading leg; (2) the presence of the second obstacle placed three steps away from the first one would increase the toe clearance in both legs and increase the obstacle-to-heel strike distance of the leading leg and (3) the toe clearance would be higher in older adults than in young adults while stepping over the second obstacle.

## Results

No failure of obstacle negotiation or fall was observed in this study in either the young or the older adult group. In addition, adding gender as a covariate in the statistical model did not alter the major findings.

### Age effects (Hypothesis #1)

We hypothesized that older adults would increase toe clearance in both legs and decrease the obstacle-to-heel strike distance of the leading leg.

1. **Walking speed during level walking (unobstructed):** Older adults (1.03 ± 0.18 m/s) walked significantly slower than younger adults (1.23 ± 0.19 m/s, p = 0.036).
2. **Spatial gait parameters (**Fig. 1**):** Significantly higher normalized toe clearances of the leading leg (F_1,17_ = 4.57, p = 0.047) and the trailing leg (F_1,17_ = 18.91, p < 0.0001) were found in older in comparison to younger adults, when either the leading or trailing leg crossed the obstacle.

**Figure 1:**
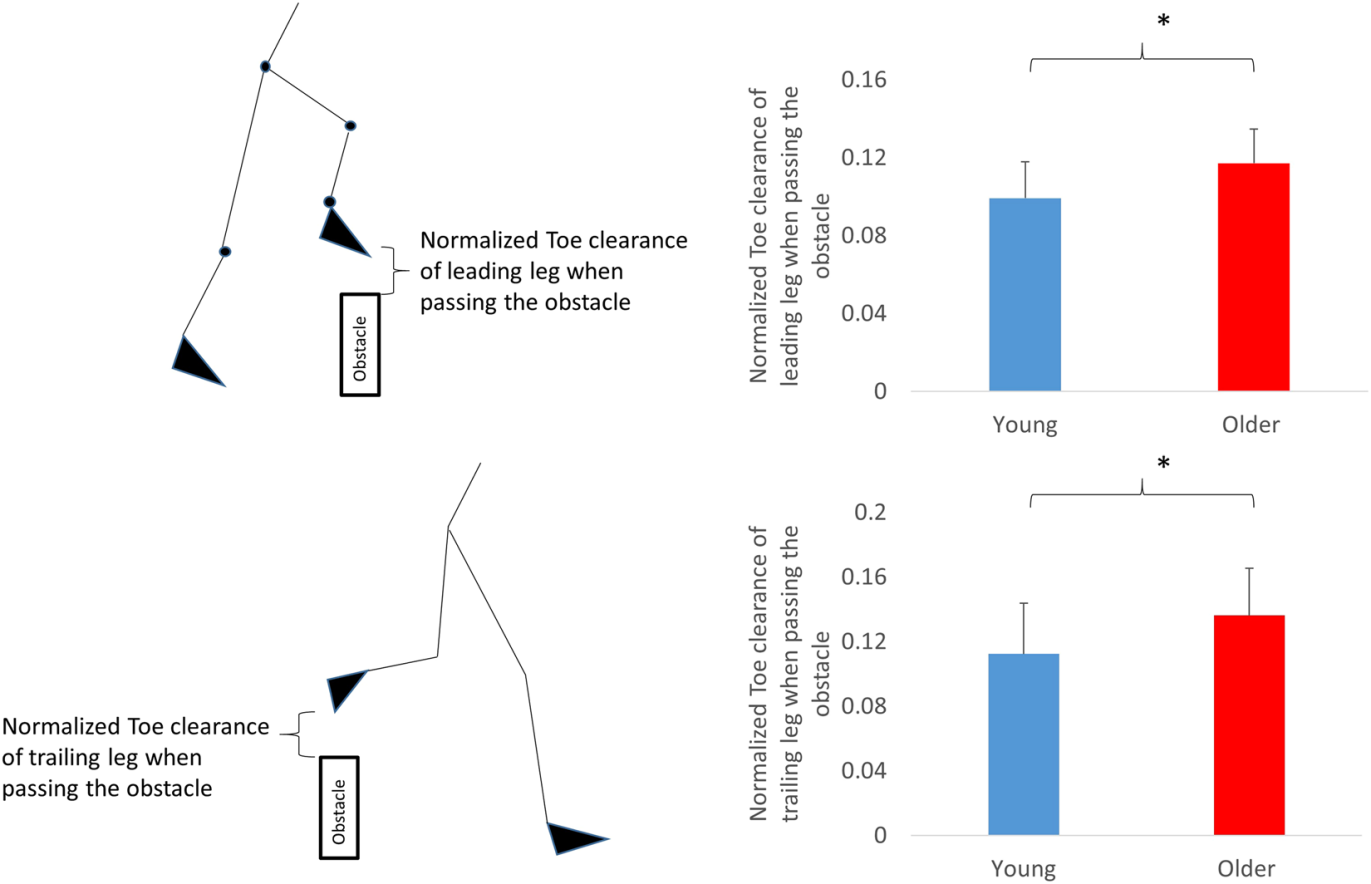
Main effect of age on multiple obstacle negotiation. * represents the significant age effect at the toe clearance of leading and trailing legs.
3. **Joint Angles (**Table 1**):** At the moment when either the leading or trailing leg crossed the obstacle, a significant age effect was observed at the hip joint of the leading leg (F_1,17_ = 10.51, p = 0.005), and of the trailing leg (F_1,17_ = 6.39, p = 0.022). In addition, a significant age effect was found at the knee joint (F_1,17_ = 4.67, p = 0.045) and at the ankle joint (F_1,17_ = 17.05, p = 0.001) at the heel-strike of the leading leg after stepping over the obstacle. Moreover, a significant age effect was observed at the knee joint (F_1,17_ = 7.29, p = 0.015) at the toe-off of the trailing leg before stepping over the obstacle. Further values are provided in Table 1.

**Table 1:** Step time (s), Normalized Step length, Joint angles (degree) at four obstacle negotiation events – toe clearance of leading leg  while stepping over the obstacle, toe clearance of trailing leg while stepping over the obstacle, Heel-Strike of the leading leg after stepping over obstacles, and Toe-Off of the trailing leg before stepping over obstacles.

Step Time (s)			Step length			
Ob1	Young	Older			Young	Older
	0.41(0.01)	0.52(0.02)	Ob1		0.32(0.07)	0.29(0.07)
Ob2	0.39(0.01)^&^	0.54(0.02)^!,&^	Ob2		0.38(0.05)^&^	0.26(0.09)^!,&^
**Toe Clearance of Leading Leg while stepping over the obstacle**						
Ob1	Hip^*,#^		Knee		Ankle	
	Young	Older	Young	Older	Young	Older
	47.22(4.86)	54.45(5.08)	86.23(13.59)	80.78(4.54)	−3.07(3.66)	−5.54(6.22)
Ob2	44.29(5.59)	52.89(5.63)	84.99(14.96)	82.49(7.58)	−1.36(4.26)	−4.28(4.89)
**Toe Clearance of trailing Leg while stepping over the obstacle**						
Ob1	Hip^*,#^		Knee^#^		Ankle^#^	
	Young	Older	Young	Older	Young	Older
	17.71(5.44)	18.64(5.13)	91.55(7.26)	96.28(7.06)	−8.05(5.78)	−7.81(5.76)
Ob2	17.71(4.79)	26.77(5.79)^!,&^	95.62(8.21)	99.94(6.46)	−11.67(8.51)	−11.77(8.89)
**Heel-Strike of the leading leg after stepping over obstacles**						
Ob1	Hip^#^		Knee^*^		Ankle^*^	
	Young	Older	Young	Older	Young	Older
	18.25(2.61)	17.27(3.85)	8.63(3.58)	5.01(4.32)	−7.12(5.30)	−12.33(4.03)^!^
Ob2	17.53(1.99)	15.24(2.94)	7.52(4.32)	4.56(4.03)	−4.33(4.26)	−14.00(3.29)^!^
**Toe-off of the trailing leg before stepping over obstacles**						
Ob1	Hip		Knee^*^		Ankle^#^	
	Young	Older	Young	Older	Young	Older
	−10.03(2.39)	−9.91(2.95)	24.35(3.16)	18.43(5.69)	8.73(4.56)	9.66(7.12)
Ob2	−10.61(2.55)	−8.43(4.73)	24.28(4.36)	20.05(7.27)	12.46(5.63)	12.30(6.84)

^*^ represents that there was a significant main effect of age and ^#^ represents that there was a significant main effect of obstacle, ^!^ represents that there was a significant difference between cell means for corresponding age group (young vs older) and ^&^ represents that there was a significant statistical difference between cell means for corresponding obstacles (Ob1 vs Ob2). Ob1: first obstacle, Ob2: second obstacle. Positive value: hip flexion, knee flexion, ankle dorsi-flexion. Negative value: hip extension, knee extension, ankle plantar-flexion.

### Obstacle effects (Hypothesis #2)

We hypothesized that the presence of the second obstacle placed three steps away from the first one would lead to an increase in toe clearance in both legs and increase the obstacle-to-heel strike distance of the leading leg.

1. **Spatial gait parameters (**Fig. 2**):** A significant effect of obstacle was found in the normalized toe clearance of the trailing leg (F_1,17_ = 15.65, p = 0.001) at the moment when the trailing leg crossed the obstacle, and in the normalized obstacle-to-heel-strike distance of the leading leg (F_1,17_ = 16.97, p = 0.001). The results showed that subjects significantly increased the toe clearance of the trailing leg and the obstacle-to-heel-strike distance of the leading leg while stepping over the second obstacle as compared to the first one.

**Figure 2:**
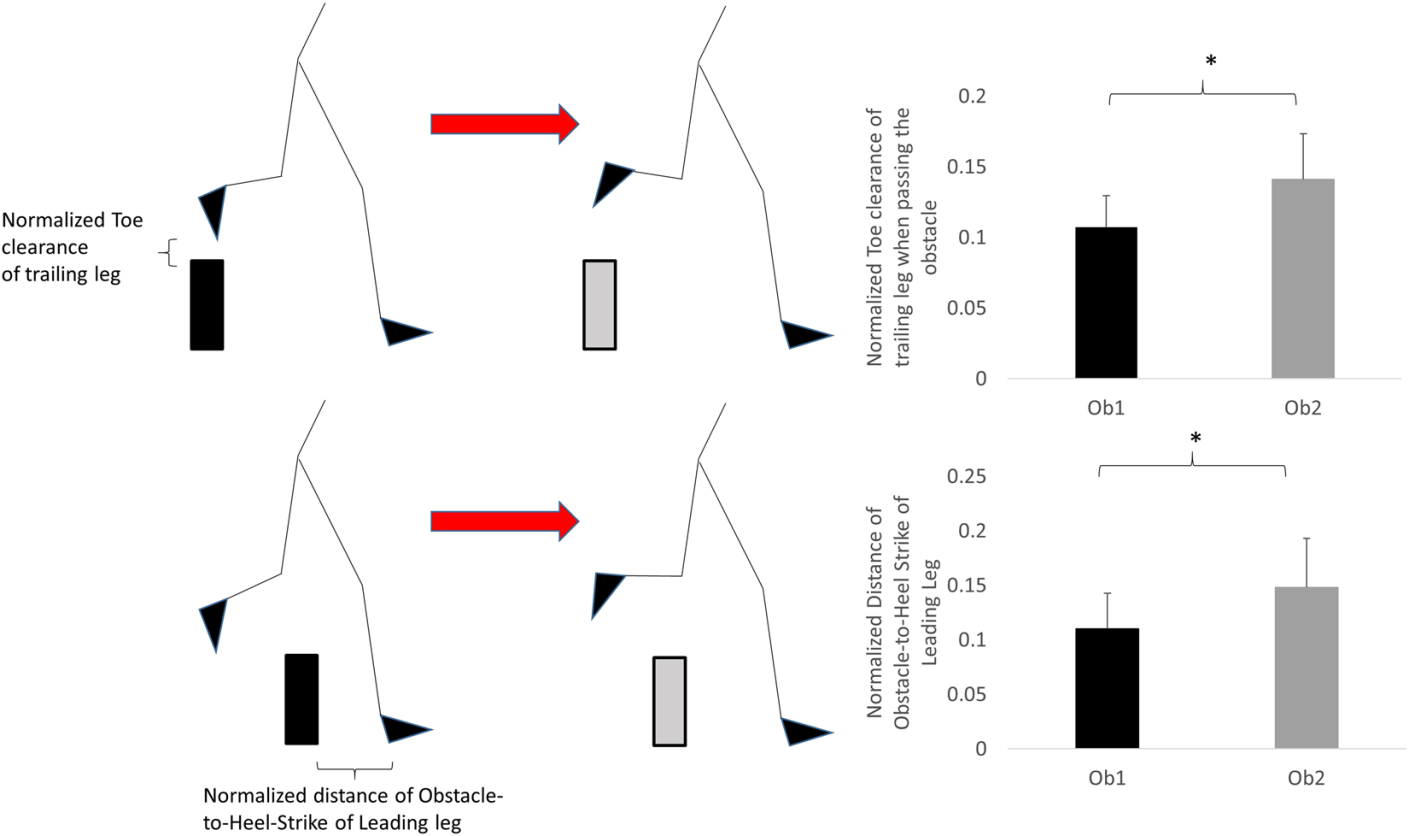
Main effect of obstacle on multiple obstacle negotiation. * represents the significant obstacle effect at the toe clearance of trailing legs and the heel-strike of leading legs.
2. **Joint Angles (**Table 1**):** A significant effect of obstacle was found at the hip joint at three events – the heel-strike of the leading leg after stepping over the obstacle (F_1,17_ = 5.57, p = 0.031), the moment when the trailing leg crossed the obstacle (F_1,17_ = 4.66, p = 0.045), and the moment when the leading leg crossed the obstacle (F_1,17_ = 4.95, p = 0.04). In addition, a significant major obstacle effect was found at the knee joint (F_1,17_ = 6.19, p = 0.024) and at the ankle joint (F_1,23_ = 10.26, p = 0.005) at the moment when the trailing leg crossed the obstacle. Finally, a significant effect of obstacle was found at the ankle joint at the toe-off of the trailing leg while stepping over the obstacle (F_1,17_ = 13.24, p = 0.002).

### The Interaction between the age effect and obstacle effect (Hypothesis #3)

The toe clearance was higher in older adults than in young adults while stepping over the second obstacle.

1. **Spatial gait parameters (**Table 1 and Fig. 3**):** A significant interaction was found in the step length (F_1,17_ = 19.90, p < 0.0001), step time (F_1,17_ = 22.77, p < 0.0001), normalized obstacle-to-heel-strike distance (F_1,17_ = 8.87, p = 0.008) at the heel-strike of the leading leg, the normalized toe clearance (F_1,17_ = 4.50, p = 0.049) at the moment when the trailing leg crossed the obstacle, and the normalized toe-off-to-obstacle distance at toe-off of the trailing leg (F_1,17_ = 5.22, p = 0.035). The post-hoc pairwise comparisons showed that younger adults had longer normalized obstacle-to-heel distances of the leading leg while crossing the second obstacle compared to the first obstacle (t(9) = −4.89, p = 0.0008). Moreover, they showed longer normalized obstacle-to-heel distances of the leading leg than those of the older adults (t(18) = 2.21, p = 0.04). The post-hoc pairwise comparisons indicated that older adults had higher normalized toe clearances of the trailing leg while crossing the second obstacle compared to the first obstacle (t(9) = −4.85, p < 0.001). Moreover, older adults showed higher normalized toe clearances of the trailing leg than young adults over both the first obstacle (t(18) = −2.51, p = 0.022) and the second obstacle (t(18) = −2.32, p = 0.032). Significant interactions were found in step time (F_1,17_ = 22.77, p < 0.001) and step length (F_1,17_ = 19.90, p < 0.001). The post-hoc pairwise comparisons revealed that step length was significantly shorter in older adults than in young adults while crossing the second obstacle. Young adults significantly decreased their step time while crossing the second obstacle in comparison to the first one (t(9) = 4.97, p = 0.001); however, older adults significantly increased their step time while crossing the second obstacle in comparison to the first one (t(9) = −2.65, p = 0.026). The post-hoc pairwise comparisons also showed that the step time was significantly longer in older adults than in young adults while crossing the second obstacle (t(18) = −2.53, p = 0.021).

**Figure 3:**
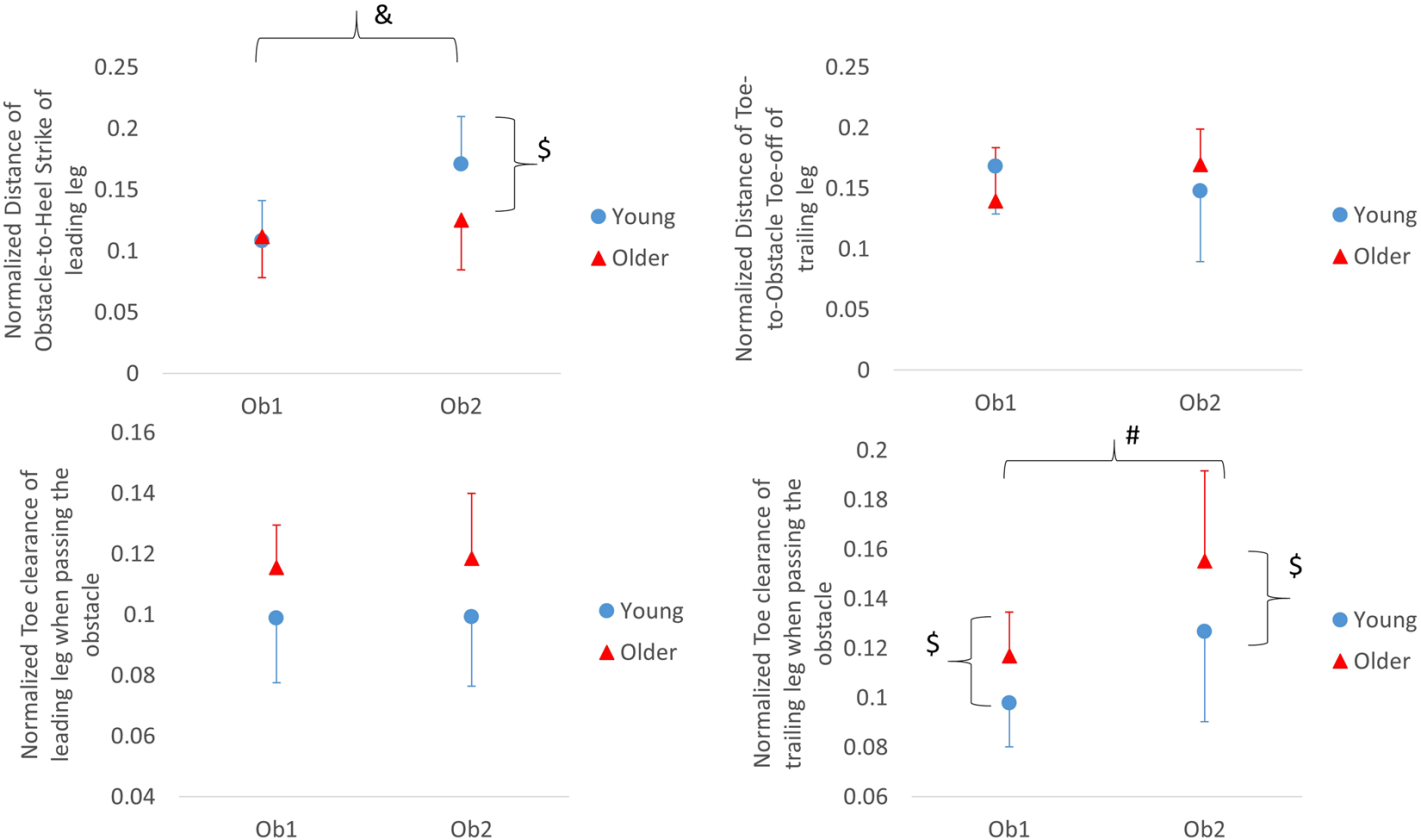
The post hoc pairwise comparisons of the marginal cell means. ^&^ represents the significant differences between two obstacles in young adults; ^#^ represents the significant differences between two obstacles in older adults; ^$^ represents the significant differences between young and older adults.
2. **Joint Angle (**Table 1**):** A significant interaction was found at the ankle joint at the heel-strike of the leading leg after stepping over the obstacle (F_1,17_ = 8.22, p = 0.011), and at the hip joint at the moment when the trailing leg crossed the obstacle (F_1,17_ = 7.89, p = 0.012). The post-hoc pairwise comparisons showed that older adults dorsi-flexed less than younger adults at the heel-strike after stepping over both obstacles (Ob1: t(18) = −5.68, p < 0.001; Ob2: t(18) = −2.47, p = 0.024) and flexed the hip joint more than younger adults while stepping over the second obstacle (t(18) = −3.81, p = 0.001).

### The Effect size

For the significant interaction between obstacle and age, the partial eta squared was 0.539 for step length, 0.573 for step time, 0.178 for the distance-to-heel-stride of leading leg, 0.091 for toe clearance of the trailing leg, 0.326 for ankle joint at the heel-strike after stepping over the obstacle, 0.17 for the hip joint at the moment when the trailing leg crossed the obstacle. The partial eta squared values revealed a moderate to large size effect^25^.

## Discussion

In the current study, we investigated the strategies involved in multiple obstacle negotiation in both healthy young and older adults when obstacles were placed three-step-length apart. We attempted to answer three questions: 1) Would age affect kinematics (joint angles and spatial gait parameters) while stepping over two obstacles? 2) Would the obstacle negotiation strategy be similar between stepping over the first obstacle and the second obstacle? 3) Would an interaction between age and obstacle effect exist? Our findings supported our hypotheses.

### Influence of Age on Multiple Obstacles Negotiation Strategy

#### Aging increased the toe clearance in both the leading and trailing legs

Previous studies have found that age affects kinematic parameters during “single” obstacle negotiation^6,7,14^. The current results extended the existing knowledge by revealing that the effect of aging also increased the toe clearance in both leading and trailing legs while stepping over “multiple” obstacles. It might be that older adults selected conservative strategies (higher toe clearance and shorter step length) to ensure sufficient leg elevation to safely step over obstacles regardless of whether there was a single or multiple obstacles^6,7,14^. Under this conservative strategy in older adults (greater vertical movement and shorter horizontal movement), the use of the hip joint was more crucial than the use of other joints due to the requirement to stabilize the movement of upper torso due to the short step length^26^. Specifically, our findings showed that older adults preferentially utilized the hip joint rather than the knee joint to raise the legs while stepping over multiple obstacles. Declines in muscle strength could be one of the reasons why older adults tended to use the proximal (hip) rather than the distal joints (knee and ankle) to negotiate multiple obstacles^27^. It has been shown that reductions in skeletal muscle strength of 20–40% occur by age 70, and this reduction in strength starts from the distal joints^28,29^. Therefore, this significant reduction of distal muscle strength forces older adults to use the hip muscles to compensate for distal muscle weakness since the power generation requirements from the hip joint tend to be easier than at the distal joints^27^.

Our findings contradict the results of a previous literature^8^, which reported no significant differences between younger and older adults in the toe clearance of the leading and trailing legs while stepping over multiple obstacles. This may be due to the difference in the interval between the two obstacles across studies. This explanation is supported by one study^22^ that showed a U-shape relationship between spatial kinematic parameters and the relative distance between two obstacles, although the study only focused on healthy young adults. The authors conclude that placing two obstacles at an interval either greater or less than two-step length apart reduces the capability of visual guidance for obstacle negotiation^22^. The visual guidance involves complex sensory processing from visual information, selective attention, and executive function^30^. Similarly, we observed that greater intervals between two obstacles reduced the capability of visual guidance in both young and older adults at the instance of crossing the obstacle in the trailing leg when the visual information was not available.

#### Aging reduced knee flexion at the heel-strike of the leading leg and the toe-off of the trailing leg

To the best of our knowledge, the study of joint motion at these two obstacle negotiation events has been limited. These two events require the body system to be stabilized for handling weight-bearing. Therefore, unlike the hip and ankle joints, which are the primary joints for generating power to move the body over obstacles, the knee joint plays an important role in stabilizing the system at these two events^31^. In the current study, the reductions in knee flexion angle at these two obstacle negotiation events in older adults might be explained by two rationales – (1) They allowed for flexibility of the other joints to absorb the impact at the heel-strike or to generate power at toe-off or (2) they were only due to the slower walking speed. For rationale #1, this strategy was supported by a previous study^32^ that showed that older adults tended to reduce their knee flexion to maintain dynamic balance during walking when encountering conditions of sensory conflict. Thus, we speculated that the reduced knee flexion at these two events observed in the current study provided the older adults with a more stable system to enable other joints to either move the body forward or stop the body and prepare for the next movement. For rationale #2, it has been shown that slowing the walking speed significantly reduces the flexion of the knee joint due to a lesser loading response at heel-strike and toe-off events^33^. In the current study, older adults significantly reduced their walking speed in comparison to young adults.

### Influence of the Second Obstacle on Multiple Obstacle Negotiation Strategy At Different Age Groups

The presence of a second obstacle in the pathway indeed influenced obstacle negotiation strategies in the current study. The proprioceptive system, specifically, might play an important role in transferring information about obstacles from the first attempt of stepping over an obstacle to the following attempts^19^. Our results provided further evidence supporting this speculation by demonstrating a longer normalized obstacle-to-heel-strike distance in the leading leg while stepping over the second obstacle than the first one in young adults. It is well documented that an increased obstacle-to-heel-strike distance results in a safer strategy of obstacle negotiation^8,9,18^. The distance of obstacle-to-heel-strike is treated with careful consideration in previous literature because a reduction in this distance could increase the likelihood of stepping down on to the obstacle^8,9,18^, which happens to older adults frequently.

Moreover, the relative interval between obstacles in the current study was set three-step-length apart. Based on a previous study^22^, the three-step-length interval between obstacles in the current study was intended to reduce the capability of visual guidance, which involves visual information search, sensory information processes, and executive function. Although we observed that longer intervals between obstacles induced higher toe clearance in older individuals, which indirectly infers the deterioration of executive function, the transfer effect between two different legs seemingly still existed in young adults. Therefore, this transfer effect between two obstacles might be provided majorly by the proprioceptive system in young adults. Our results supported that the transfer effect happened in young adults not only when the interval between obstacles was two steps apart (subjects used the same leg to step over two obstacles)^23^ but also when the interval between obstacles was three steps apart (subjects used the different leg to step over two obstacles). Similarly, Harris *et al*. (2001) found that the ability to discriminate the roughness of a surface can be transferred to the neighboring fingers and to corresponding fingers of the contralateral side^34^. It is possible that effective neuronal inter-limb coupling is important for efficient human movement^35^ and this speculation might be applied to the situation of multiple obstacle negotiation. However, such a transfer effect was not found in older adults while stepping over the second obstacle in the current study. This could be due to the deterioration of proprioceptive function in older adults^12^; therefore, this transfer effect could not happen between legs when the interval between obstacles was not long enough.

In summary, our study clearly demonstrated that older adults use different strategies to perform multiple obstacle crossing, specifically while stepping over a second obstacle three steps away from the first one. Thus, the multiple obstacles protocol (e.g. varied locations between obstacles) may be a technique for detecting the risk of falling in older adults. However, there were three limitations of this study. First, our sample size was only 10 subjects per group for data analysis. For the outcomes that reached significance between the effect of obstacle and age in the current study, our partial eta squared results indicated a moderate-to-large effect size^25^. Second, we did not perform any cognitive assessment to examine the deterioration of executive function in older adults. Third, we did not perform any visual acuity test. Although we asked subjects to report their visual acuity status verbally, lack of visual acuity might hinder the true visual guidance capability when performing multiple obstacle negotiation. In future studies, a simple visual acuity test could be used to exclude subjects if their scores are below 20/20 with corrected glasses. Those limitations should be considered in future work.

## Methodology

### Subjects

Fifteen healthy young adults (10 female and 5 male; age 27.33 ± 6.29 years; height 1.72 ± 0.09 m; and weight 71.7 ± 15.72 kg) and ten healthy older adults (7 female and 3 male; age 66.70 ± 5.21 years; height 1.69 ± 0.06 m; and weight 75.33 ± 12.82 kg) participated in this study. For pairing purposes, we reduced the sample size of the young adults from 15 to 10. We randomly picked 10 young adults using “randi” function in MATLAB R2011a (Mathworks, Natick, MA). Therefore, ten healthy young adults (6 females and 4 males; age 28.1 ± 7.08 years; height 1.71 ± 0.09 m; and weight 75.33 ± 12.82 kg) and ten healthy older adults participated in this study. All older subjects were living independently and did not require a walking aid (cane or walker) during the testing session. Subjects were excluded from the study if they had a history of visual or vestibular deficits or any neurological disorder. Before data collection, we verbally asked subjects “Can you see the surroundings clearly when you walk straight, without glasses.” If subjects could not see the surroundings clearly, visual acuity was corrected by wearing glasses. Subjects also filled in a health questionnaire to determine whether they had sustained any falls in the previous year. If they had had any falls in the past year, they were excluded from the study. This study was carried out in accordance with relevant guidelines and regulations of the University of Nebraska Medical Center Institutional Review Board. In addition, all experimental protocols were approved by the University of Nebraska Medical Center Institutional Review Board (IRB # 304-14-EP). All subjects signed informed consent documents before experiments began.

### Experimental materials

**Figure 4:**
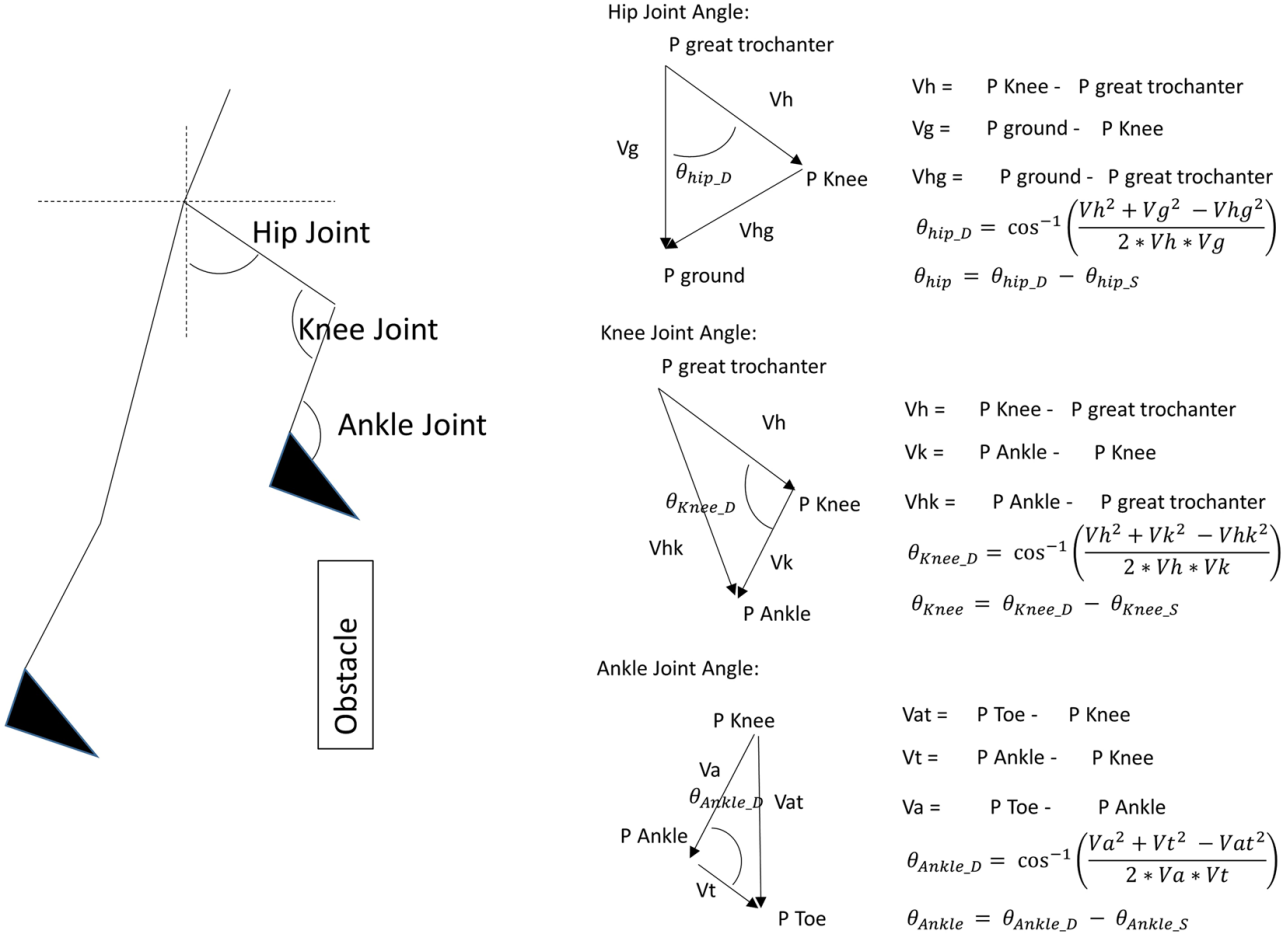
The definition of joint angles. The joint angles were set to 0 when standing on anatomical neutral standing position. Positive value: hip flexion, knee flexion, and ankle dorsi-flexion. Negative value: hip extension, knee extension, and ankle plantar-flexion. *θ*_*hip_D*_, *θ*_*knee_D*_, *θ*_*Ankle_D*_,: angles in walking conditions, *θ*_*hip_S*_, *θ*_*knee_S*_, *θ*_*Ankle_S*_,: angles in standing condition.

**Figure 5:**
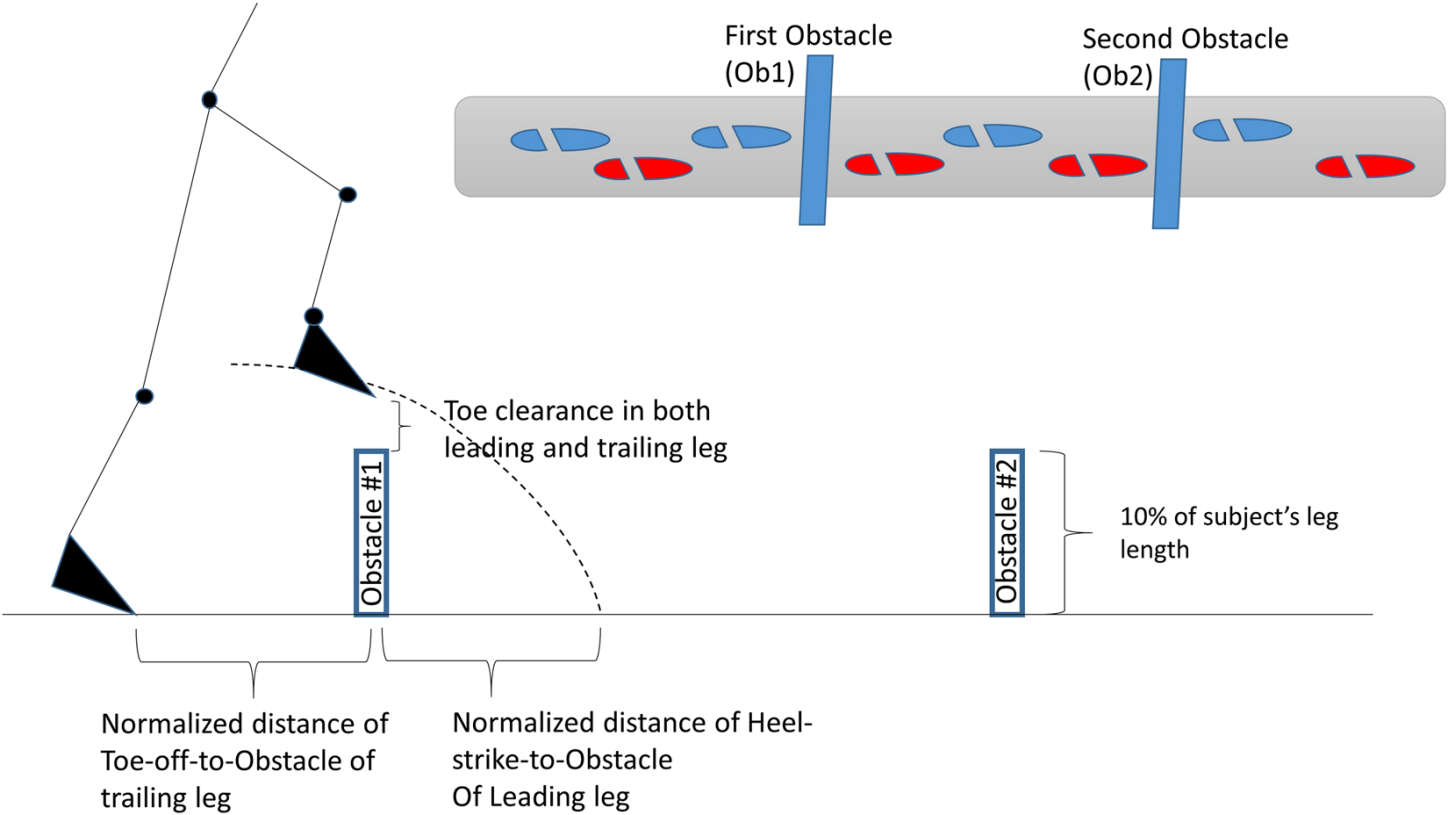
The experimental design and the dependent variables.

Spherical retro-reflective markers and an infra-red eight-camera motion capture system (Qualisys AB, Gothenburg, Sweden) were used to collect three-dimensional kinematic data using Qualisys Tracker Manager (QTM) software (Qualisys AB) at 100 Hz. Retro-reflective markers were placed on the greater trochanter of the femur, lateral epicondyle of the femur, lateral malleoli, toe (second metatarsophalangeal joint), and heel of both legs. Two markers were fixed at the top edge of each obstacle. Four obstacle negotiation events were investigated as follows: toe clearance of the leading leg (vertical distance from the toe marker to the height of the obstacle while crossing an obstacle), toe clearance of the trailing leg (vertical distance from the toe marker to the height of the obstacle while crossing an obstacle), the moment when the heel of the leading leg contacted the floor (heel-strike) after stepping over an obstacle, and the moment when the toe of the trailing leg lifted off the floor (toe-off) before stepping over an obstacle. All kinematic parameters and joint angles were determined using a custom-written MATLAB R2011a program (Mathworks, Natick, MA). The step time was the time from the heel-strike of the leading leg to the toe-off of the trailing leg. The step length was the horizontal distance between the moment of the heel-strike of the leading leg and the moment of the toe-off of the trailing leg. All joint angles were set at 0 degrees in anatomical neutral standing position and the joint angles in walking conditions were offset by the joint angle from standing trial. Joint angles were calculated according to Hamill *et al*.^36^ as shown in Fig. 4. Participant traversed a walkway (0.8 m × 6 m active area). Two identical obstacles made of PVC (shape: cylinder, height: 0.6 m, radius: 0.02 m) were placed three-step-length apart with height set at 10% of each subject’s leg length (Fig. 5).

### Experimental Protocol

Once the informed consent was completed, subjects performed two walking tasks consisting of unobstructed (level ground walking) and obstructed (stepping over two obstacles) conditions. First, subjects were instructed to walk straight at a self-selected pace five times for 6 meters in the unobstructed condition. Then, the average stride length and average preferred walking speed were calculated. The average step length was used to calculate the appropriate interval between the two obstacles and the interval between the starting point of the traverse and the first obstacle. The first obstacle was placed approximately three steps away from the starting point of each subject, and the second obstacle was placed approximately three steps away from the first one. We chose to adjust the inter-obstacle distance according to the regular step length of each subject in order to present a similar challenge to all subjects. Subjects were instructed to perform the obstructed condition five times. We limited the numbers of trials to reduce potential for adaptation to stepping over the second obstacle that could potentially occur in as few as 5 attempts^37^. The subjects were free to choose which leg they began walking with. Subjects rested for 1 minute between trials in order to eliminate any learning effects from the previous trial.

### Statistical Analysis

A mixed repeated measures ANCOVA (2 age groups × 2 obstacles, with gender as a covariate factor) was used to investigate the effect of aging and the effect of two obstacles for each dependent variable. The significance level was set at 0.05. When a significant interaction effect was determined, post-hoc pairwise comparisons were used. Statistical analysis was completed in SPSS 18.0 (IBM Corporation, Armond, NY). To understand the effect size we used the partial eta squared method. The partial eta squared has been widely used for measuring the effect size and was at least 0.138 for large effect size, 0.059 for moderate effect size, and 0.01 for small effect size^25^.
